# Lung Ultrasound Evaluation of Aeration Changes in Response to Prone Positioning in Acute Respiratory Distress Syndrome (ARDS) Patients Requiring Venovenous Extracorporeal Membrane Oxygenation: An Observational Study

**DOI:** 10.7759/cureus.55554

**Published:** 2024-03-05

**Authors:** Jiaping Huai, Xiaohua Ye

**Affiliations:** 1 Department of Critical Care Medicine, Affiliated Jinhua Hospital, Zhejiang University School of Medicine, Jinhua, CHN; 2 Department of Gastroenterology, Affiliated Jinhua Hospital, Zhejiang University School of Medicine, Jinhua, CHN

**Keywords:** prognostic indicator, venovenous extracorporeal membrane oxygenation, acute respiratory distress syndrome, prone positioning, lung ultrasound

## Abstract

Background: Prone positioning (PP) has been proven to be a beneficial approach in enhancing survival outcomes for patients with severe acute respiratory distress syndrome (ARDS) who need venovenous extracorporeal membrane oxygenation (V-V ECMO) support. The study utilized bedside lung ultrasound (LUS) to evaluate changes in lung aeration caused by PP in ARDS patients receiving V-V ECMO.

Methods: This retrospective single-center study involved adult ARDS patients requiring V-V ECMO. The assessment of LUS involved examining specific dorsal lung regions, encompassing 16 areas, during three pre-defined time points: baseline (10 minutes prior), three-hour PP positioning, and 10-minute post-supine repositioning, all within the initial three days. Based on the oxygenation response to PP, patients were categorized into responder and non-responder groups. The primary outcome was LUS score changes during the initial three-day period. Secondary outcomes examined the impact of PP on the partial pressure of oxygen (PaO_2_)/fraction of inspired oxygen (FiO_2_) (P/F) ratio, V-V ECMO weaning success, length of ICU stay, and hospital survival.

Results: Among the enrolled patients (27 in total), 16 were responders and 11 were non-responders. In the responder group, the global LUS score underwent a significant reduction from 26.38 ± 4.965 at baseline to 20.75 ± 3.337 (p < 0.001) after the first PP session, which further decreased to 15.94 ± 2.816 (p< 0.001) after three days. However, no significant differences were observed among PP non-responders. The oxygenation reaction yielded comparable results. There was a significant correlation between the duration of daily PP and the reduction in global LUS score among PP responders (r = -0.855, p < 0.001). In cases where the global LUS score decreased by > 7.5 after three days of PP, the area under the receiver operating characteristic curve (AUROC) for predicting ECMO weaning success was 0.815, while it was 0.761 for predicting hospital survival.

Conclusion: LUS has the potential to predict the response to PP and evaluate the prognosis of ARDS patients with V-V ECMO, although more studies are demanded in the future.

## Introduction

Acute respiratory distress syndrome (ARDS) is a frequent clinical condition that continues to be a major factor in the intensive care unit (ICU) mortality despite advancements in treatment approaches [1]. The effectiveness of prone positioning (PP) in improving outcomes for individuals with ARDS has been well established [2]. When lung-protective mechanical ventilation fails to prevent hypoxia or hypercapnia in severe cases of ARDS, the use of venovenous extracorporeal membrane oxygenation (V-V ECMO) support may be considered [3,4]. Previous evidence has shown that integrating PP into adult patients with severe ARDS requiring V-V ECMO was associated with improved survival outcomes [5]. PP not only improves gas exchange by reducing ventilation/perfusion mismatch and minimizing ventilator-induced lung injury (VILI) through promoting homogeneous parenchymal aeration [6], but also provides additional benefits such as enhanced lung recruitability, improved secretion drainage, and reduced strain on the right ventricle [7]. However, in certain patients, PP may be ineffective or potentially harmful, as it can lead to accidental extubation, catheter dislodgement, pressure ulcers, and hemodynamic instability [8]. Therefore, evaluating the effectiveness of PP in ARDS patients undergoing V-V ECMO is of utmost importance in order to determine the potential benefits and predict the likelihood of positive outcomes.

Ultrasound is gaining popularity in the ICU due to its non-invasive nature, bedside availability, ease of repetition, and reproducibility [9]. Lung ultrasound (LUS) is a reliable tool that provides accurate information on lung condition, aeration, recruitment, perfusion, and morphology, offering valuable insights into the state of the lungs [10,11]. Numerous studies have emphasized the value of LUS in assessing how lung aeration responds to PP in intubated patients with ARDS [12-14]. In comparison to chest radiography or computed tomography, LUS minimizes the risks related to intrahospital transfer and exposure to radiation [15]. However, the effectiveness of LUS in accurately predicting patient response to PP varied across different research studies [12,13,16].

The main aim of this study was to evaluate the influence of PP on lung aeration in patients with ARDS requiring V-V ECMO within the initial three days and to investigate whether alterations detected by LUS during PP could serve as prognostic indicators for ECMO withdrawal and hospital survival. Our hypothesis was that better lung aeration during PP would be associated with increased rates of ECMO withdrawal and improved survival.

## Materials and methods

This prospective study was conducted at the medical ICU of the Affiliated Jinhua Hospital, Zhejiang University School of Medicine, Zinhua, China, from April 5, 2019, to September 29, 2023. The study protocol was approved by the Jinhua Central Hospital Medical Ethics Review Committee (approval number: 20222730201). Informed consent was acquired from each patient or their legal surrogate decision-maker prior to the commencement of any study procedures.

Patient selection

All eligible patients who met the inclusion criteria and required mechanical ventilation were consecutively enrolled in the study. Patients with a definite diagnosis of ARDS based on the Berlin definition [17] were included. V-V ECMO was implemented in patients with ARDS who met one of the specified criteria despite optimal mechanical ventilation and recommended additional therapies: (i) partial pressure of oxygen (PaO_2_)/fraction of inspired oxygen (FiO_2_) (P/F) ratio < 50 mmHg for > 3 hours, or (ii) P/F ratio < 80 mmHg for > 6 hours, or (iii) arterial pH < 7.25 with arterial carbon dioxide partial pressure (PaCO_2_) ≥ 60 mmHg for > 6 hours with respiratory rate of 35 breaths/minute and mechanical ventilation settings adjusted to maintain plateau pressure (P_plat_) ≤ 32 cm H_2_O [4]. Exclusion criteria were as follows: (1) < 18 years old, (2) pregnancy.

ECMO management and PP sessions

V-V ECMO cannulas were skillfully inserted by experienced intensivists, primarily utilizing femoral-jugular access. Our ECMO center had the capability to convene the entire ECMO team within 30 minutes. In cases where patients were critically unstable, our ECMO team would transport a portable system to primary-care hospitals, perform the device implantation at the patient's bedside, and subsequently transfer them to our facility. Our ECMO team operated 24/7, guaranteeing constant availability for patient care. The ECMO flow rate was adjusted to achieve an ECMO flow/cardiac output [18], while the sweep gas flow was regulated to secure PaCO_2_ levels < 45 mmHg. The fraction of inspired oxygen in the circuit was modified to maintain arterial oxygenation levels ≥ 90%. In patients without severe bleeding, intravenous unfractionated heparin was administered to target a partial thromboplastin time 1.5 times the upper normal limit. Daily evaluations were performed using the EOLIA (ECMO to Rescue Lung Injury in Severe ARDS) clinical and physiological criteria to assess the potential for ECMO weaning [19].

The attending physician exercised discretion when deciding to implement PP for patients on V-V ECMO, considering factors such as the P/F ratio value, extensive lung consolidation, and the difficulty of weaning from ECMO. The duration of planned PP sessions was set for a minimum of 16 hours [20]. Responders were defined as patients who demonstrated a P/F change of ≥ +20 mmHg following PP, whereas non-responders were identified as those with a P/F change < 20 mmHg [21].

LUS measurements

All examinations were conducted using a 2-5 MHz curved array probe (FUJIFILM SonoSite, Bothell, Bothell, Washington, United States) paired with an M-Turbo ultrasound machine. Daily LUS assessments were conducted during the initial three days of recruitment, contingent upon the successful weaning of the patient from ECMO. The LUS examination was conducted at three specific time points: 10 minutes before PP (pre-PP), three hours after PP (post-PP), and 10 minutes after resuming the supine position (post-supine) following the initial prone session of each day. The examination areas were determined using body markers: paravertebral line, scapular line, and posterior axillary line. Each side of the back was divided into three regions, resulting in nine examination areas. Within each region, three equal areas were identified, totaling eight points on a single side (excluding the point covered by the scapular bone). Overall, there were 16 points examined for both sides.

The sonographic indications of lung aeration were categorized into four groups: A-line alone or in conjunction with fewer than three B lines (0 points: normal aeration pattern); B lines detected in less than 50% of the pleural line (1 point: B1 aeration pattern); B lines observed in over 50% of the pleural line (2 points: B2 aeration pattern); complete loss of aeration indicating lung consolidation (3 points: C aeration pattern). The global LUS score was calculated by adding up the scores from the 16 sonographic lung regions. The LUS ranged from 0, indicating a normal aeration pattern, to 48, representing a complete loss of aeration [12]. Two certified ICU expert physicians, proficient in critical LUS, anonymously and blindly assessed and scored each LUS video recording during a retrospective evaluation.

Outcomes

Primary outcomes were successful ECMO weaning as well as ICU and hospital survival. Successful ECMO weaning was described as being ECMO-free and surviving for a minimum of 48 hours following decannulation. Unsuccessful weaning was defined as the inability to remove the ECMO device within 48 hours due to ongoing respiratory failure or mortality during ECMO support, necessitating the need for re-cannulation.

The primary outcome was the change in global LUS score from pre-PP on day 1 to post-PP on day 3. The secondary outcome was P/F ratio, length of ECMO support, ECMO weaning success ratio, length of mechanical ventilation, ICU length of stay, hospital length of stay, and survival between responders and non-responders.

Statistical analysis

IBM SPSS Statistics for Windows, Version 22.0 (Released 2013; IBM Corp., Armonk, New York, United States) was employed for the statistical analysis. The data were summarized as either mean ± standard deviation or median values along with interquartile ranges (IQR). The Mann-Whitney U test was employed to assess the differences between the two groups. Differences among the variables pre-PP, post-PP, and post-supine were assessed using either repeated measures ANOVA or Friedman's test. The computation of the r correlation coefficient was based on Pearson's correlation test. The area under the receiver operating characteristic curve (AUROC) was computed to determine the accuracy of different variables in predicting V-V ECMO weaning success and hospital survival. All statistical tests were two-tailed, and p values < 0.05 were considered statistically significant.

## Results

**Figure 1:**
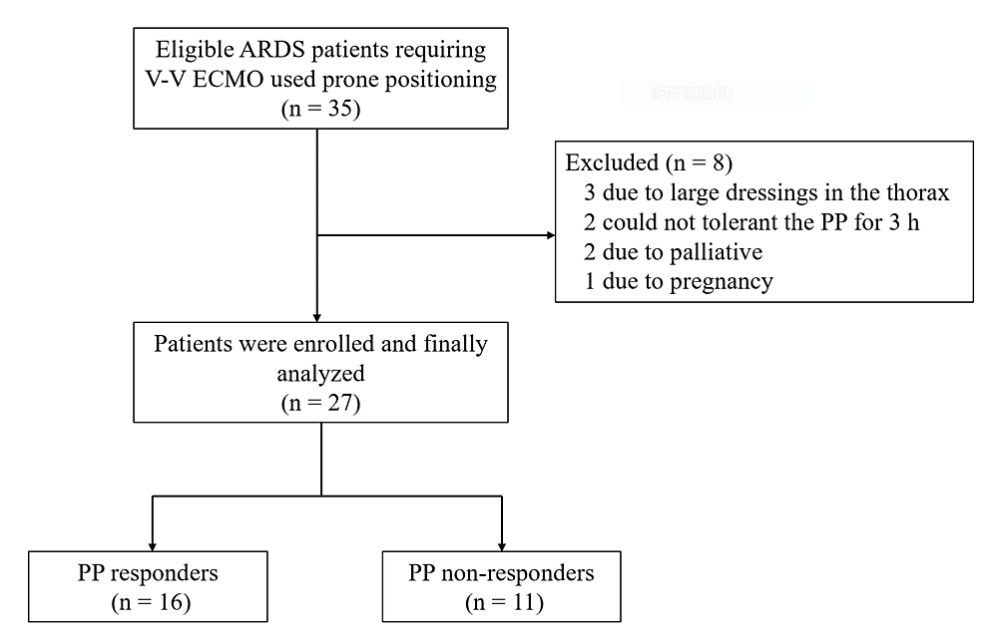
Flowchart of the study ARDS, acute respiratory distress syndrome; V-V ECMO, venovenous extracorporeal membrane oxygenation; PP, prone positioning

A total of 35 consecutive patients were initially enrolled in the study. However, eight patients were excluded from the analysis, which included three patients with large dressings in the thorax, two patients who experienced hemodynamic alterations and could not tolerate PP for three hours, two patients under palliative care, and one pregnant patient. Consequently, the final analysis consisted of 27 patients (Figure 1), and Table 1 provides a detailed description of the baseline characteristics of the study population.

**Table 1:** Comparison of baseline characteristics and outcomes between PP responders and non-responders Data are expressed as number (percentages), mean ± standard deviation, or median (interquartile). Driving pressure was calculated as plateau pressure–total positive end-expiratory pressure. SOFA, sequential organ function assessment; PP, prone positioning; V-V ECMO, venovenous extracorporeal membrane oxygenation; PEEP, positive end-expiratory pressure; FDO_2_, fraction of delivered oxygen in the sweep gas.

Characteristics and Outcomes	PP Responders (n = 16)	PP Non-responders (n = 11)	p-value
Age (years)	57.3 ± 7.7	60.8 ± 8.9	0.279
Male (n, %)	9 (56.3)	6 (54.5)	0.581
Body mass index (kg/m^2^)	27.6 ± 2.9	29.2 ± 2.3	0.135
SOFA score	9.5 ± 1.9	10.5 ± 2.0	0.222
Comorbidities			
Hypertension (n, %)	8 (50)	7 (63.6)	0.484
Diabetes (n, %)	6 (37.5)	6 (54.5)	0.381
Immunodepression (n, %)	3 (18.8)	2 (18.2)	0.970
Chronic kidney disease (n, %)	4 (25)	3 (27.3)	0.895
Chronic cardiac disease (n, %)	5 (31.3)	3 (27.3)	0.824
Malignancy (n, %)	1 (6.3)	0 (0)	0.398
PP before V-V ECMO (n, %)	13 (81.3)	10 (90.9)	0.488
Time from V-V ECMO to the first PP (days)	4.3 ± 2.2	3.6 ± 1.9	0.457
Mechanical ventilation settings before the first PP			
Tidal volume (ml/Kg)	5 ± 0.6	5.2 ± 0.6	0.462
PEEP (cmH_2_O)	13.9 ± 1.2	14.5 ± 0.6	0.163
Driving pressure (cmH_2_O)	15.7 ± 1.4	16 ± 1.4	0.568
Respiratory system compliance (mL/cmH_2_O)	19.4 ± 3.4	18.6 ± 2.56	0.504
Inspired fraction of oxygen (%)	74.7 ± 11.9	79.1 ± 11.8	0.352
V-V ECMO settings before the first PP			
V-V ECMO blood flow (L/min)	5.4 ± 0.5	5.1 ± 0.6	0.172
Sweep gas flow (L/min)	3.4 ± 0.5	3.2 ± 0.4	0.280
FDO_2_ (%)	100 (90-100)	100 (90-100)	0.497
Duration of PP (hours)	16.9 ± 0.9	17.1 ± 1.3	0.611
Outcomes			
Duration of V-V ECMO (days)	17.6 ± 3.8	23.1 ± 3.6	0.001
Duration of mechanical ventilation (days)	29.2 ± 4.9	36.3 ± 5.4	0.002
V-V ECMO weaning (n, %)	13 (81.3)	3 (27.3)	0.005
ICU length of stay (days)	38.2 ± 5.2	52.8 ± 5	< 0.001
Hospital length of stay (days)	45.4 ± 4.6	56.4 ± 4.7	< 0.001
Hospital survival (n, %)	12 (75)	3 (27.3)	0.014

Out of the 27 patients, 59.3% (16 out of 27) experienced a P/F change of ≥ +20 mmHg following the initial PP therapy, categorizing them as the PP responders group. Conversely, 40.7% (11 out of 27) of the patients had a P/F change < 20 mmHg, assigning them to the PP non-responders group. No significant complications were encountered during the positioning procedures. The complications were recorded in Table 2, and the complications were similar to patients with PP responders and non-responders. Additionally, baseline characteristics showed no significant differences between the two groups.

**Table 2:** Complications during the positioning procedures Data are presented as n (%). PP, prone positioning

Complications	PP Responders (n = 16), n (%)	PP Non-responders (n = 11), n (%)
Renal	5 (31.3)	3 (27.3)
Hemorrhagic	3 (18.8)	2 (18.2)
Neurologic	1 (6.3)	0 (0)
Infectious	1 (6.3)	1 (9.1)
Cardiovascular	2 (12.5)	1 (9.1)
Pulmonary	1 (6.3)	0 (0)
Pressure sores	2 (12.5)	1 (9.1)
Other	3 (18.8)	2 (18.2)

Lung aeration scores and P/F ratio between PP responders and non-responders

**Figure 2:**
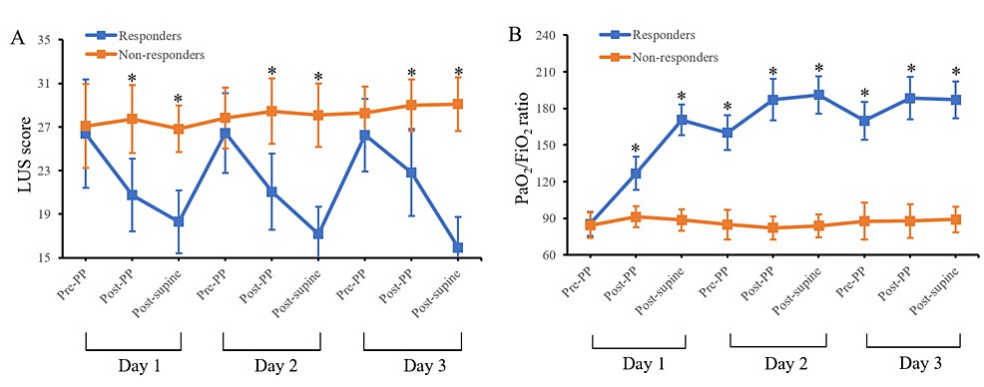
Evolution of measured variables Data are expressed as mean ± standard deviation; *p < 0.05 between the two groups The graphs show a comparison of LUS (A) and PaO_2_/FiO_2_ ratio (B) between patients with PP responders (blue symbols and lines) and non-responders (red symbols and lines). PP, prone positioning; PaO_2_, partial pressure of oxygen; FiO_2_, fraction of inspired oxygen; LUS, lung ultrasound

Following the initial PP session, the LUS score decreased significantly from 26.7 ± 4.5 to 21.8 ± 5 (p < 0.001). Subsequently, the LUS score further decreased to 21.3 ± 7.1 (p = 0.001) after three days. In PP responders, significant reductions were observed in global LUS scores over the initial three days (from 26.4 ± 5 to 15.9 ± 2.8, p < 0.001), while no significant changes were noted in PP non-responders. Patients who responded to PP had a notably lower LUS score after the first PP session compared to non-responders (18.3 ± 2 vs. 26.8 ± 2.1, p < 0.001), even though their pre-PP global LUS scores were similar (p = 0.69) (Figure 2A).

The improvement in oxygenation among both PP responders and non-responders mirrored the changes observed in the global LUS score. However, the PP responder group showed a significant improvement in the P/F ratio after three days compared to PP non-responders (89.2 ± 10.4 vs. 187.1 ± 15.1, p < 0.001) (Figure 2B). Additionally, only patients who responded to PP demonstrated a significant enhancement in the post-supine P/F ratio on all three days (all p < 0.05).

Correlation of changes in LUS scores, P/F ratio, and mean daily PP duration

**Figure 3:**
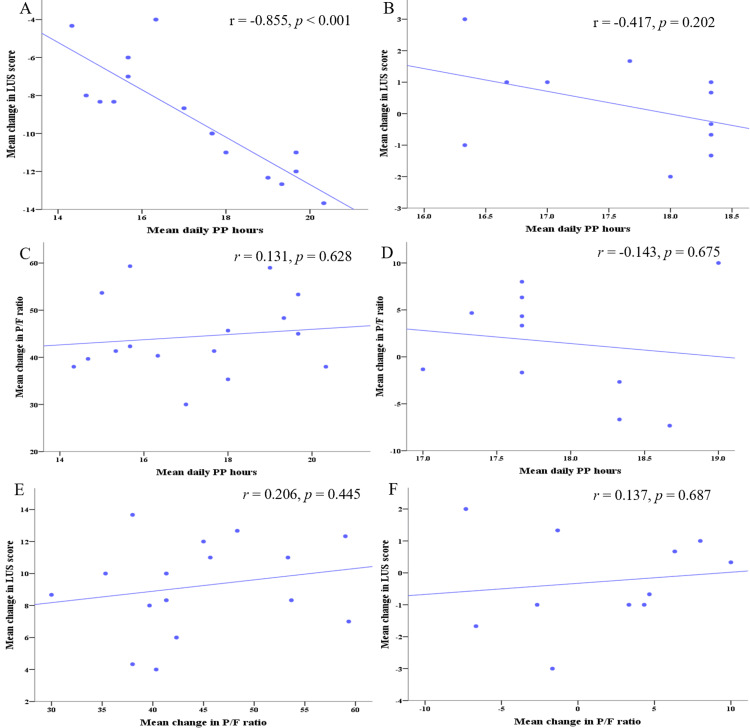
Scatter plot shows the correlation of mean daily duration of PP, LUS score, and PaO2/FiO2 ratio Correlation of mean daily duration of PP and LUS score of the three days in PP responders (A) and PP non-responds (B); Correlation of mean daily duration of PP and PaO_2_/FiO_2_ ratio of the three days in PP responders (C) and PP non-responds (D); Correlation of mean change in LUS score and PaO_2_/FiO_2_ ratio of the three days in PP responders (E) and PP non-responds (F) PP, prone positioning; PaO_2_, partial pressure of oxygen; FiO_2_, fraction of inspired oxygen; LUS, lung ultrasound

The daily durations of PP for responders and non-responders on days 1, 2, and 3 were recorded as 17.1 ± 2.6 vs. 17.5 ± 1.6 h, 17.2 ± 3 vs. 17.4 ± 1.5 h, and 17.4 ± 2.1 vs. 18 ± 1.5 h, respectively (*p* > 0.05). Within the first three days, a significant correlation was found in the PP responder group between the mean daily duration of PP and the decrease in global LUS score (r = -0.855, p < 0.001) (Figure 3A). Conversely, no significant correlation was observed in the PP non-responder group (r = -0.417, p = 0.202) (Figure 3B). However, the mean daily duration of PP has no correlation with the change of P/F ratio in either PP responders or non-responders (r = 0.131, p = 0.628; r = -0.143, p = 0.675) (Figure 3C, Figure 3D). Furthermore, no significant associations were found between the mean changes in global LUS score and P/F ratio for both PP responders and non-responders (r = 0.206, p = 0.445; r = 0.137, p = 0.687) (Figure 3E, Figure 3F).

Prediction of ECMO weaning success and hospital survival among patients

Of the 27 patients, 17 (63%) were effectively weaned off from V-V ECMO. The average duration of V-V ECMO therapy for all patients was 19.9 ± 4.6 days. PP responders had a shorter duration of ECMO and mechanical ventilation treatment compared to non-responders (17.6 ± 3.8 vs. 23.1 ± 3.6 days, p = 0.001; 29.2 ± 4.9 vs. 36.3 ± 5.4 days, p = 0.002, respectively) (Table 1). A decrease in global LUS score > 7.5 at post-supine after three days demonstrated the highest AUROC of 0.815 (95%CI: 0.655-0.975) for predicting ECMO weaning success, with a sensitivity of 68.8% and specificity of 90.9%. Despite these findings, the AUROC of P/F ratio for predicting successful ECMO weaning after PP on the second day was 0.724 (95%CI: 0.515-0.934), with a sensitivity of 68.8%, specificity of 81.8%, and a cut-off value greater than 19 (Table 3).

**Table 3:** AUROC predicting the ECMO weaning success and hospital survival with changes of LUS score and P/F ratio Δ-global LUS, change in global LUS after the prone positioning session; Δ-P/F ratio, change in P/F ratio after the prone positioning session; AUROC, area under the receiver operating characteristic curve; ECMO, extracorporeal membrane oxygenation, LUS, lung ultrasound score; P/F, partial pressure of oxygen (PaO_2_)/fraction of inspired oxygen (FiO_2_)

Variable	AUROC (95% CI)			p-value			Cut-off			Sensitivity			Specificity	
	ECMO weaning success	Hospital survival		ECMO weaning success	Hospital survival		ECMO weaning success	Hospital survival		ECMO weaning success	Hospital survival		ECMO weaning success	Hospital survival
Day 1														
Δ-global LUS	0.804 (0.625-0.983)	0.731 (0.522-0.939)		0.008	0.043		< -5.5	< -5.5		73.3	66.7		90.9	83.3
Δ-P/F ratio	0.642 (0.413-0.871)	0.594 (0.366-0.823)		0.217	0.407		> 17.5	> 17.5		81.3	80		63.6	58.3
Day 2														
Δ-global LUS	0.795 (0.619-0.972)	0.772 (0.594-0.951)		0.01	0.017		< -0.5	< -0.5		93.8	93.3		54.5	50
Δ-P/F ratio	0.724 (0.515-0.934)	0.708 (0.503-0.914)		0.051	0.067		> 19	> 19		68.8	66.8		81.8	75
Day 3														
Δ-global LUS	0.75 (0.561-0.939)	0.703 (0.501-0.905)		0.03	0.075		< -7.5	< -7.5		68.8	66.7		81.8	75
Δ-P/F ratio	0.514 (0.289-0.74)	0.547 (0.323-0.772)		0.902	0.678		> 8.5	> 22.5		68.8	86.7		45.5	33.3
Whole 3 days														
Δ-global LUS	0.815 (0.655-0.975)	0.761 (0.579-0.943)		0.006	0.022		< -7.5	< -7.5		68.8	66.7		90.9	83.3
Δ-P/F ratio	0.615 (0.43-0.871)	0.603 (0.379-0.826)		0.191	0.367		> 20	> 20		75	73.3		63.6	58.3

The overall hospital survival rate was 55.6% (15/27), with a higher survival rate observed in the PP response group at 75% (12/16) compared to the PP non-response group at 27.3% (3/11; p = 0.014). All patients who were successfully weaned from ECMO had favorable hospital discharge results, except for one patient who experienced multiple organ failure and died within one week after ECMO withdrawal. The AUROC values for predicting hospital survival based on LUS score were 0.731, 0.772, 0.703, and 0.761 on day 1, day 2, day 3, and for the entire three-day period, respectively. After a three-day period, Youden's J statistic determined the optimal global LUS cut-off to be < -7.5, with a sensitivity of 66.7% and specificity of 83.3%. The highest AUROC of 0.708 (0.503-0.914) for predicting hospital survival was observed when the P/F ratio increased by 19 after PP on day 2, with sensitivity and specificity rates of 66.8% and 75%, respectively (Table 3).

## Discussion

The primary findings of this study on LUS evaluation of aeration changes in ARDS patients undergoing V-V ECMO and PP can be summarized as follows: (1) Improved global LUS score and P/F ratio in PP responders compared to PP non-responders; (2) Among PP responders, there was a significant correlation between mean daily PP duration and a reduction in global LUS score, whereas no correlation was observed among PP non-responders. Additionally, there was no significant correlation between mean daily PP duration and P/F ratio in both PP responders and non-responders; (3) Reduction in the global LUS score exceeding 7.5 after three days of PP was found to be indicative of successful weaning from V-V ECMO.

ARDS is characterized by severe respiratory failure arising from hypoxia, affecting up to 10% of ICU patients and frequently necessitating the application of mechanical ventilation [22]. In ARDS patients who continue to suffer from severe hypoxemia despite conventional ventilation strategies, V-V ECMO emerges as a highly effective salvage therapy [3]. Multiple studies have reported favorable outcomes in oxygenation and respiratory system compliance when PP is employed in ECMO-treated patients [23,24]. However, some studies have not demonstrated a significant reduction in mortality associated with the use of PP during ECMO [25,26]. Furthermore, patients subjected to PP tend to experience prolonged stays in the ICU and require an extended duration of ECMO support [27]. Therefore, evaluating the response to PP in ECMO patients assumes crucial importance.

In recent years, there has been a growing interest among researchers in studying changes in lung aeration during ventilation with PP. The assessment of lung reaeration in intubated ARDS patients has often involved the use of computed tomography (CT) scans [28]. Additionally, the use of transesophageal echocardiography (TEE) has been implemented to monitor changes in density area in patients during PP [29]. However, both CT and TEE are not easily obtainable in clinical practice. In contrast, LUS provides a bedside, real-time, non-invasive, functional, reproducible, and radiation-free imaging technique for evaluating lung reaeration. We observed a significant reduction in the overall LUS score among patients who underwent PP. This finding aligns with a previous study that demonstrated higher changes in aeration scores, as assessed by LUS, in the responder and survivor group of PP patients [12]. Thus far, there have been limited effective indicators to predict the prognosis of PP in initial instances. The ability to directly quantify lung aeration during PP confers a decisive advantage in prognostication, as compromised aeration represents a key pathophysiological factor. Our findings can facilitate the early identification of patients at a heightened risk of PP failure, enabling more intensive monitoring and tailored therapeutic interventions to mitigate the risk of severe complications during the procedures.

Despite a significant association between changes in the global LUS score and PP duration among PP responders, no correlation was observed between PP duration and oxygenation changes, regardless of responder status. This finding aligns with previous research suggesting that the benefits of PP are independent of oxygenation and decarboxylation responses, and may be more closely related to a reduction in VILIs [30]. Earlier studies have reported that approximately 30% of patients do not exhibit an oxygenation response to PP [31], and while an increase in the P/F ratio has been observed during PP, it often decreases upon returning to the supine position [32]. Another physiological study involving CT scans and electrical impedance tomography (EIT) evaluations in coronavirus disease 2019 (COVID-19) patients yielded similar results. It found significant alveolar recruitment in the dorsal region and de-recruitment in the ventral region during PP, but no significant correlation was observed between overall or regional lung recruitment and oxygenation. The response in oxygenation during PP is influenced by a balance between lung recruitment and de-recruitment, as well as changes in lung perfusion. Therefore, evaluating the extent of dorsal recruitment based solely on oxygenation response may not be appropriate. The conversion of images into digital formats (semiquantitative) is crucial for effectively assessing lung aeration. LUS offers an attractive semiquantitative approach for describing regional aeration, rather than relying solely on global lung air content. Changes in LUS scores have been shown to correlate with CT measurements of lung aeration during the initiation of antimicrobial therapy in patients with ventilator-associated pneumonia [33]. Additionally, in successful spontaneous breathing trials, changes in aeration measured by LUS scores can accurately predict post-extubation distress [10]. Our study demonstrates the relationship between changes in lung aeration, as assessed by LUS, and the response to PP in ARDS patients requiring V-V ECMO.

LUS has been identified as a valuable indicator for assessing lung parenchymal injury and has shown an independent association with 28-day mortality in patients with shock [34]. Consistent findings have been reported in other studies, where individuals with higher LUS scores demonstrated a significantly increased risk of adverse clinical outcomes [35]. The research found that a LUS score of > 24 points demonstrated the highest accuracy in predicting ICU admission or death. Our study demonstrated that a reduction in the global LUS score of > 7.5 points within a three-day period following PP predicted successful weaning from V-V ECMO and hospital survival. Consequently, the evaluation of LUS has the potential to identify ARDS patients requiring V-V ECMO who have a better prognosis.

Our study has limitations. First, the LUS evaluation was performed at a single time point, and we did not assess the response of PP in other sessions. Second, LUs has methodological heterogeneity. Although our study's outcomes are in line with the existing literature, it is essential to recognize potential methodological disparities that warrant attention. These could include differences in protocol type, the number of regions evaluated, the specific transducer utilized, and machine settings. Third, we did not incorporate CT imaging or advanced respiratory monitoring methods like EIT to examine lung aeration and perfusion. Consequently, we are unable to provide data or make definitive conclusions about the extent of global and regional lung overdistension, as well as modifications in lung perfusion, that occurred during the study. Moreover, due to the lack of prospective control over mechanical ventilation settings, V-V ECMO settings, and hemodynamics, their influence on the observed outcomes remains uncertain. Lastly, the relatively small sample size in our study underscores the importance of conducting larger-scale studies to validate the promising results.

## Conclusions

LUS emerges as a valuable tool for monitoring aeration changes in the lung-dependent region during PP in ARDS patients receiving V-V ECMO support. Utilizing LUS to assess these aeration changes enables the prediction of oxygenation response to PP. Notably, a decrease in the global LUS score > 7.5 following PP may aid in identifying patients who are likely to successfully wean off V-V ECMO and achieve favorable hospital outcomes.
